# Molecular evolution of TRPC4 regulatory sequences supports a role in mammalian thermoregulatory adaptation

**DOI:** 10.7717/peerj.19697

**Published:** 2025-07-08

**Authors:** Robert S. Cornman

**Affiliations:** Fort Collins Science Center, U.S. Geological Survey, Fort Collins, CO, United States of America

**Keywords:** Adaptation, Molecular evolution, Positive selection, Evolutionary rates, Mammals, Thermoregulation, Transient receptor potential channels, Nonshivering thermogenesis, Brown adipose tissue

## Abstract

**Background:**

Proteins encoded by the canonical transient receptor potential (Trpc) gene family form transmembrane channels involved in diverse signal-transduction pathways. *Trpc4* has been shown necessary for the induction of nonshivering thermogenesis (NST) in mice, a key component of which is thermogenic brown adipose tissue (BAT). In bats, T*rpc4* exhibited diversifying selection within exons encoding regulatory binding sites of TRPC4.

**Methods:**

To assess whether diversification of these regulatory sequences mirrors the diversification of mammalian thermoregulatory strategies, the ratio of nonsynonymous to synonymous substitutions (ω) was estimated for multiple tetrapod outgroups and eutherian orders. Four questions were addressed: (1) Did the ancestral eutherian *Trpc4* diverge under positive selection from nonplacental mammals that lack BAT? (2) Did *Trpc4* subsequently become more constrained in descendant eutherian clades? (3) In eutherian clades that subsequently lost BAT by inactivation of the thermogenin gene *Ucp1*, did *Trpc4* become less constrained? (4) Does the evolutionary rate of *Trpc4* differ between quantitatively more heterothermic mammal orders (bats and rodents) relative to quantitatively less heterothermic outgroups (carnivores, artiodactylids, and primates)?

**Results:**

Coincident with the advent of BAT, *Trpc4* evolutionary rate increased significantly in ancestral eutheria after their divergence from nonplacental mammals but a branch-site model did not support a rate class ω > 1 along that branch. In descendant eutherian mammals, *Trpc4* became far more constrained, with an evolutionary rate less than half that of tetrapod clades lacking NST, a pattern was not seen in other Trp channel genes. Intensifying selection in descendent eutherian mammals was further supported with the RELAX program, which also indicated reduced constraint on *Trpc4* in clades that have secondarily lost BAT. However, no consistent pattern was identified within mammalian orders with strong variation in heterothermy: evidence of increased evolutionary rate was again found in bats for *Trpc4* as well as homologs it directly binds in heteromeric membrane channels (*Trpc5* and *Trpc1*), yet all rodent Trpc genes had low evolutionary rates. Evolutionary rates of *Trpc4* and *Trpc1* in bats were consistent with relaxed constraint whereas bat *Trpc5* experienced diversifying selection. Most variation among tetrapod TRPC4 sequences lies within an  85 amino-acid window that is functionally uncharacterized. Sequence alignments demonstrated that the TRPC4 β isoform, which lacks a portion of the C-terminal regulatory region, originated in basal eutherians but appears to be lost in many tip lineages. Collectively, the data indicate that the C-terminal region of TRPC4 has responded to selection on NST thermoregulation during the diversification of eutherian mammals. The drivers of increased diversification of *Trpc4* and interacting genes in bats remain to be determined.

## Introduction

The transient receptor potential (Trp) ion channels are proteins that form nonselective, gated transmembrane channels, permitting the flow of Ca^2+^ and other ions upon activation by regulating molecules (Ramsey, Delling & Clapham, 2006). In vertebrates, the ‘canonical’ TRP subfamily (TRPC) of proteins participate in diverse processes including cardiac and neurological function. They also function in the transduction of environmental stimuli such as thermoregulation, proprioception, chemoreception and the perception of pain (Minke & Cook, 2002). Major regulatory molecules that are known to alter TRPC channel activity include calmodulin, inositol triphosphate receptors (ITPRs), phospholiplase C, stromal interaction molecule 1 (STIM1), and G-protein coupled receptors, as well as several scaffolding proteins and small molecules such as diacylgycerol and phosphatidylinositol 4,5-bisphosphate (PIP_2_). These molecules primarily interact with various C-terminal residues of TRPCs that project interior to the plasma membrane (Kim et al., 2019; Chen et al., 2020).

*Trpc4* has been shown by Zhou et al. (2023) to be essential in mice for non-shivering thermogenesis (NST), a suite of behavioral and metabolic responses to core temperature change that are regulated by warm-sensing neurons (WSNs) in the preoptic area of the hypothalamus (Tan et al., 2016). A key mechanism effecting NST is metabolic heat generation by brown adipose tissue (BAT), although thermogenesis can also occur in muscle (Nowack et al., 2017). BAT thermogenesis is mediated by thermogenin, also known as uncoupling protein 1 (UCP1), and is considered an ancestral trait of eutherian mammals (Keipert et al., 2024). BAT thermogenesis is important during acute cold stress (particularly in small-bodied mammals) and for postnatal survival, and may play a role in the regulation of hibernation in some species (Hashimoto et al., 2002; Heldmaier, Klaus & Weisinger, 1990; Bienboire-Frosini et al., 2023).

*Trpc4* has separately been identified as a positive-selection candidate in bats (order Chiroptera) and exhibits a higher rate of diversification of the regulatory protein-binding region in that clade compared to related mammalian orders (Cornman, 2024). In light of the functional analyses of Zhou et al. (2023), this increased diversification of *Trpc4* in bats may be associated with thermoregulatory divergence within that clade. Numerous studies have documented ecologically important differences in the thermoregulatory physiology of bats, including the use of hibernation and torpor as well as tolerance of changes in core body temperature (Tb) while active (*e.g.*, Cryan & Wolfe 2003; Stawski, Willis & Geiser, 2014; Czenze & Dunbar, 2017; Czenze et al., 2022; Rubalcaba et al., 2022). More generally, NST exhibits strong evolutionary lability in eutherian mammals, as evidenced by the independent pseudogenizations of *Ucp1* in several eutherian groups, which has been associated with Pliocene climate change and increased body size (Gaudry et al., 2017). Thus, ecological diversification of mammalian thermoregulation might be reflected in protein evolution of TRPC4 at various phylogenetic scales if such changes are important modulators of NST.

Support for the hypothesis that diversifying selection on TRPC4 regulatory sequences is related to thermoregulatory adaptation in mammals can be evaluated by comparing evolutionary rates of *Trpc4* and interacting genes in clades with different thermoregulatory strategies. One possible comparison is among vertebrate lineages prior to and since the advent of NST, for example by comparing predominantly ectothermic taxa (amphibians and nonavian sauropsids), taxa that thermoregulate without BAT-mediated NST (birds), and taxa that thermoregulate using BAT-mediated NST, among other mechanisms (eutherian mammals). This comparison would reveal whether *Trpc4* evolutionary rates changed in a manner consistent with functional divergence and subsequent purifying selection due to recruitment into NST-regulating genetic pathways. A complementary comparison is made possible by the independent pseudogenizations of *Ucp1* noted above. Assuming the *Trpc4*-dependence of NST in mice is characteristic of mammals generally, evolutionary rates within the regulatory region of TRPC4 may increase following *Ucp1* loss due to the elimination of that functional constraint. An additional comparison is suggested by the pronounced effect of body size on core body temperature variation (Tb) within a species (Boyles et al., 2013). Small mammals experience a wider range of Tb than larger mammals in general, and useful generalizations can be made at the order level as to relative body size and importance of NST without neglecting the importance of adaptations within particular lineages. For example, taxa exhibiting high Tb variation are common within rodents (order Rodentia) and bats, whereas various carnivores (order Carnivora) are prominent among taxa with low variation in Tb (Boyles et al., 2013). These order-level differences allow potential associations between the evolutionary rate of *Trpc4* and the level of variation in Tb (heterothermy) to be explored. Importantly, while functional overlap may exist between genetic regulation of NST and the prolonged shifts in Tb caused by hibernation and torpor, there is as yet no basis for predicting a correlation between use of these latter strategies and *Trpc4* evolutionary rate.

A complication of this analytical framework is that TRPC channels assemble as tetramers and may be heteromeric (*i.e.,* containing more than one type of TRPC protein) and thus Trpc genes may not evolve independently. TRPC4-containing channels in mouse neurons are predominantly heteromers with TRPC5 or TRPC1 (Bröker-Lai et al., 2017; Kollewe et al., 2022), and *Trpc5* expression has itself been linked to physiological responses to temperature (Zimmerman et al., 2011; Bernal et al., 2021). In fact, functionally significant amino-acid variation affecting non-NST temperature response has been identified in the C-terminal regulatory region of *Trpc5* (Ptakova et al., 2022), the phylogenetically closest paralog of *Trpc4* (Chen et al., 2020). In contrast to *Trpc1*, *Trpc4*, and *Trpc5*, proteins encoded by Trpc gene family members *Trpc3*, *Trpc6*, and *Trpc7* form predominantly homomeric channels in neurons (Kollewe et al., 2022). Including other Trpc genes in a comparative analysis would therefore allow patterns unique to *Trpc4* to be differentiated from patterns that are common to many genes compared across a given phylogeny. The same reasoning applies to structurally similar genes of other Trp subfamilies that have identified roles in transducing temperature signals in various contexts. *Trpm2* is of particular note as it acts as a homeostatic regulator of elevated temperature (as opposed to reduced temperature) in warm-sensitive neurons (Kamm et al., 2021; Song et al., 2016). Recent work has implicated adiposal expression of *Trpa1*, *Trpv2*, and *Trpm2* in the control of BAT thermogenesis at the source (Sun, Uchida & Tominaga, 2017; Lv et al., 2023; Benzi et al., 2024), but roles in the homeostatic activation or maintenance of NST responses within warm-sensitive neurons themselves have not been reported. Thus, while the present study hypothesizes neuronal control of evolutionary variation in NST phenotypes, manifested by changes in TRPC4 in heteromeric channels, regulatory changes in thermogenic tissues themselves are a reasonable alternative.

In summary, this study investigates *Trpc4* evolutionary rates with respect to NST at different phylogenetic scales. Evolutionary rates are estimated for a discrete, unambiguously defined regulatory region of sufficient length and conservation to be informative of selection pressures acting on it. Where statistical support of rate heterogeneity is found, additional statistical models are applied to differentiate positive selection and relaxed constraint as explanatory factors. *Trpc4* evolutionary rates are then interpreted within a larger context of interacting and non-interacting Trpc homologs as well as structurally similar Trp family members with potentially overlapping functions.

## Methods

Predicted transcripts were downloaded from the ortholog browser of the National Center for Biotechnology Information (NCBI) for each gene and each taxonomic group. NCBI ortholog assignments are performed for high-quality reference genomes based on reciprocal sequence similarity to model genes as well as microsyntney of local gene order (refer to https://www.ncbi.nlm.nih.gov/kis/info/how-are-orthologs-calculated/), and are considered authoritative for the objectives of this study. The major phylogenetic bins within vertebrates for which sequence was accessed were amphibians, avian sauropsids (class Aves), non-avian sauropsids (lizards, turtles, snakes, and their relatives), eutherian mammals, and marsupials. Orthologs for two monotreme species (*Ornithorhynchus anatinus* and *Tachyglossus aculeatus*) were also accessed but after inspection were not combined with marsupials for most analyses because the two groups aligned poorly for several genes, which would have substantially reduced the number of codons available for analysis. However, monotremes were included in the branch-specific test of *Trpc4* divergence in basal Eutheria (see Results), as they aligned unambiguously and represented important variation for rate estimation. Within eutherian mammals, sequences were downloaded for the orders Chiroptera (bats), Artiodactyla (even-toed ungulates and cetaceans, synonymous with Cetartiodactyla (*sensu* Hassanin et al., 2012), Primates, Rodentia (rodents), and Carnivora (carnivores). These are the major mammalian orders for which the number of high-quality gene annotations are highest (refer to Genes database of NCBI), and thus best-suited for order-level evolutionary rate comparisons. Fish were not included in the analysis as most Trpc genes are duplicated within that clade, consistent with ancestral polyploidization (Von Niederhäusern et al., 2013). Due to fluctuation in taxonomic nomenclature used by NCBI, the following species pairs are considered synonymous: *Neogale vison* and *Neovison vison* (synonymous also with *Mustela vison*); *Lagenorhynchus obliquidens* and *Sagmatias obliquidens*; *Microtus fortis* and *Alexandromys fortis; Physeter catodon* and *Physeter macrocephalus*.

Nucleotide alignments were initially generated with MAFFT v. 7.490 (Katoh et al., 2002) and edited with BioEdit v. 7.7.1 (Hall, 1999) by first trimming upstream of start codons using the coordinates annotated by NCBI for each transcript. Alignments were then collapsed, translated, and re-aligned at the amino-acid level with ClustalW v. 1.81 (Thompson, Gibson & Higgins, 2003) before reverting to nucleotide sequence to trim alignments downstream of stop codons. Ambiguously aligned regions (*e.g.*, gapped sequence, low-complexity sequence, or splice-site variants) were manually trimmed between the nearest unambiguously aligned codons, and sequences that were substantially incomplete in the studied region were removed. Analyzed alignments are available in File S1, and by policy are also made available in a USGS data release (Cornman, 2025).

Gene family members were dispersed in the studied genomes (*i.e.,* they do not occur as tandem arrays that may be more prone to mis-annotation or mis-assembly) and no indication of gene conversion between paralogs was observed in the alignments (*i.e.,* tracts of sequence similar between paralogs in the same taxon but dissimilar to orthologs from related taxa). All Trp gene alignments were confirmed to begin with the conserved TRP domain (the “TRP Box” depicted in Fig. 1.4 of Rosasco & Gordon, 2017) that marks the transition between transmembrane channel domains and the intracellular tail of the protein (Duan et al., 2018; Vinayagam et al., 2018). The initial residues of the domain are highly conserved within each Trp family (Rosasco & Gordon, 2017) and readily identifiable. Regulatory binding sites, alternative splice sites, and other sequence features of the region were extracted from (Tang et al., 2001; Mery et al., 2002; Odell, Van Helden & Scott, 2008; Miehe et al., 2010; Yuan et al., 2003) as well as the Genome Viewer utility of NCBI (Rangwala et al., 2021). As previously reported by Frankenberg et al. (2011), *Trpc2* was found to be inconsistently annotated due to its close genomic proximity to and past conflation with *Xndc1* and was not analyzed here. A full-length alignment and dendrogram of these proteins were made for selected species to further confirm their orthology (Fig. S1). The sequences were amino-acid aligned with Mega v. 11 (Kumar et al., 2018) and a neighbor-joining tree was generated using the JTT exchange matrix and the default gamma distribution of rate variation.

Draft guide trees for evolutionary rate analysis were generated using approximately 15 kb of mitochondrial sequence downloaded from NCBI for available taxa. Mitochondrial sequences were aligned without regard to gene features with MAFFT and then trimmed of the poorly aligned control region. Trees were generated using the neighbor-joining algorithm in Mega with the maximum composite likelihood substitution model and with rate variation among sites modeled by the default gamma distribution. Taxa used in Trp gene alignments that lacked sufficient mitochondrial sequence were inserted manually into Newick-formatted trees based on established taxonomic classifications (Hassanin et al., 2012; Agnarsson et al., 2011; Blanga-Kanfi et al., 2009; Hassanin et al., 2021; Zurano et al., 2019). Guide trees were unrooted with ape (Paradis, Claude & Strimmer, 2004) prior to use and are available in File S1.

Protein evolutionary rates ω were estimated with the codeml program of PAML v. 4.9j (Yang, 2007) under several codon models. To qualitatively compare trends across genes for the various tetrapod clades and mammalian orders, single ω estimates were calculated for each gene-clade combination without rate variation among codon sites or among branches (NSsites = 0, Model = 0). For analyses explicitly parameterizing branch-level rate variation within an alignment, rate classes were marked for each branch in the tree (all branches with the same class designation share a common ω estimate) and model settings changed to NSsites = 0 and Model = 2. The statistical significance of branch-level rate variation for specific comparisons was based on the relative likelihoods of the null model, which had one or two branch rates depending on the hypothesis tested, and an alternative model with one additional rate class (see Results for details). The test statistic was twice the difference in ln(likelihood) of the two models assuming a *χ*^2^ distribution with one degree of freedom (Yang, 2007), with α set to 0.05. Note that codeml likelihoods are not based on a single most-likely ancestral sequence reconstruction but instead are weighted across all ancestral sequence probabilities reconstructed by the codeml algorithm (Yang, 1998; Anisimova, 2012); ancestral sequences are not specified by the user.

Significant changes in ω under PAML models (see Results) were further evaluated with evolutionary rate models of the HyPhy package (Pond, Frost & Muse, 2004) that explicitly model proportions of sites assigned to rate classes representing positive selection or relaxed selection (*i.e.,* branch-site models). aBSREL (Smith et al., 2015) was used to explicitly test a model of positive selection on *Trpc4* in basal Eutheria, as distinct from relaxed selection, as it requires one of three ω classes to be greater than one on the foreground branch for a nonzero proportion of sites. RELAX (Wertheim et al., 2015) was used to explicitly model change in constraint in descendent eutherian mammals relative to evolutionary rates estimated for the basal eutherian branch. The null model under this framework estimates the proportion of sites in a user-designated number of ω classes that may be less than or greater than one, but are constant across the phylogeny. The alternative model estimates a ‘relaxation’ or ‘intensification’ factor k that distinguished two different sets of ω estimates for foreground *versus* background branches of the phylogeny. For k significantly less than one, the foreground ω classes are closer to one than in background (*i.e.,* both purifying and positive selection are weaker); if k is significantly greater than 1, foreground ω classes are further from one compared with background branches (both purifying and positive selection are stronger). RELAX was used to evaluate whether changes in ω following *Ucp1*-inactivation were more consistent with relaxation or increased positive selection. Both RELAX and BUSTED (Murrell et al., 2015) were used to distinguish relaxed selection from positive diversifying selection on Trpc genes in bats. BUSTED is similar to RELAX in that three ω classes are assumed by default and are the same for all branches under the null model but differ on foreground branches under the tested model. The highest rate class is capped at one under the null model, whereas it may exceed one in the tested model; positive diversifying selection is inferred if the tested model is significantly more likely and a nonzero proportion of sites have an ω > 1. BUSTED differs from aBSREL in that all foreground branches are treated as a single partition of the data whereas aBSREL tests positive diversifying selection on each branch separately and is therefore less useful for testing pervasive change in a large section of a tree. Note RELAX was run with the default number of rate classes (3) for all analyses except the comparison of descendent eutherian mammals to the basal eutherian branch, which used only two because higher numbers resulted in at least one ω > 1 for the basal eutherian branch, in conflict with the aBSREL results (see Results).

Model outputs are summarized in File S2 and the PAML control file is given in File S1. *P*-values were adjusted for testing multiple genes or alignments for each question, if applicable, as shown in File S2, using the Benjamin-Hochberg option of the p.adjust function in R (R Core Team, 2018).

## Results

### *Trpc4* evolutionary rate was elevated at the origin of NST

**Figure 1:**
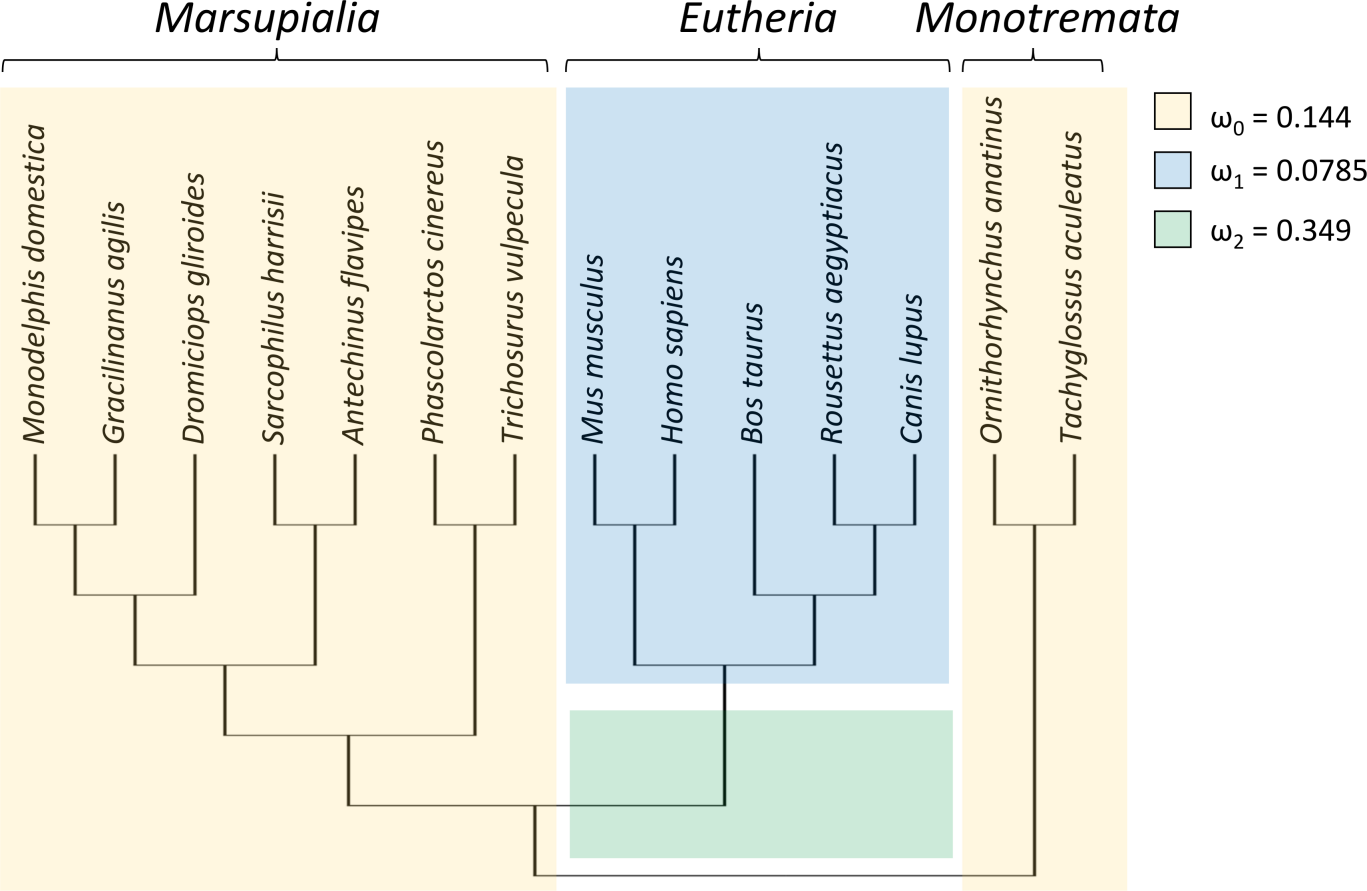
Taxa and phylogeny used in evolutionary rate tests on the canonical transient receptor potential 4 (TRPC4) regulatory region at the base of Eutheria. Evolutionary rates are modeled as the parameter ω, the ratio of nonsynonymous to synonymous substitutions along branches of a phylogeny. In the alternative hypothesis, branches were assigned to one of three rate classes as indicated by color and the estimated values of those rate classes are shown. This model was significantly more likely than a null model in which ω_2_ was set equal to ω_1_, *i.e.,* only two rate classes were assumed. A subsequent branch-specific test of positive selection at a subset of protein sites, as opposed to relaxed selection, was also significant for the branch denoted in green. The common names corresponding to the listed scientific names are as follow: gray short-tailed opossum (*Monodelphis domestica*), agile gracile opossum (*Gracilinanus agilis*), monito del monte (*Dromiciops gliroides*), Tasmanian devil (*Sarcophilus harrisii*), yellow-footed antechinus (*Antechinus flavipes*), koala (*Phascolarctos cinereus*), common brushtail possum (*Trichosurus vulpecula*), house mouse (*Mus musculus*), human (*Homo sapiens*), cow (*Bos taurus*), Egyptian fruit bat (*Rousettus aegyptiacus*), dog (*Canis lupus*), platypus (*Ornithorhynchus anatinus*), and short-beaked echidna (*Tachyglossus aculeatus*).

To test whether the evolutionary rate of *Trpc4* increased in basal eutherian mammals, a branch-specific test was performed with codeml with three branch classes (Fig. 1). In the null model, marsupials and monotremes were assigned one common evolutionary rate (ω_0_) and eutherian mammals a second evolutionary rate (ω_1_); two rates were modeled instead of one to reflect the variation among those clades (see below). The alternative (tested) hypothesis specified that the branch leading to eutherian mammals evolved at a third rate (ω_2_) that was greater than the other two rates. The data strongly supported an increased divergence rate along the basal branch of Eutheria (*P* = 4.271E−07), with ω_2_ = 0.349 compared to ω_0_ = 0.144 and ω_1_ = 0.0785. An explicit test of positive selection on this branch (*i.e.,* a nonzero proportion of sites with ω > 1) conducted with aBSREL was not significant (*P* = 0.210), however. Thus, the data confirm an increased evolutionary rate of TRPC4 regulatory sequences coincident with the evolution of BAT in Eutheria but do not definitively demonstrate the increase was due to positive selection rather than relaxed selection.

### *Trpc4* evolutionary rates became more constrained after the advent of NST

**Figure 2:**
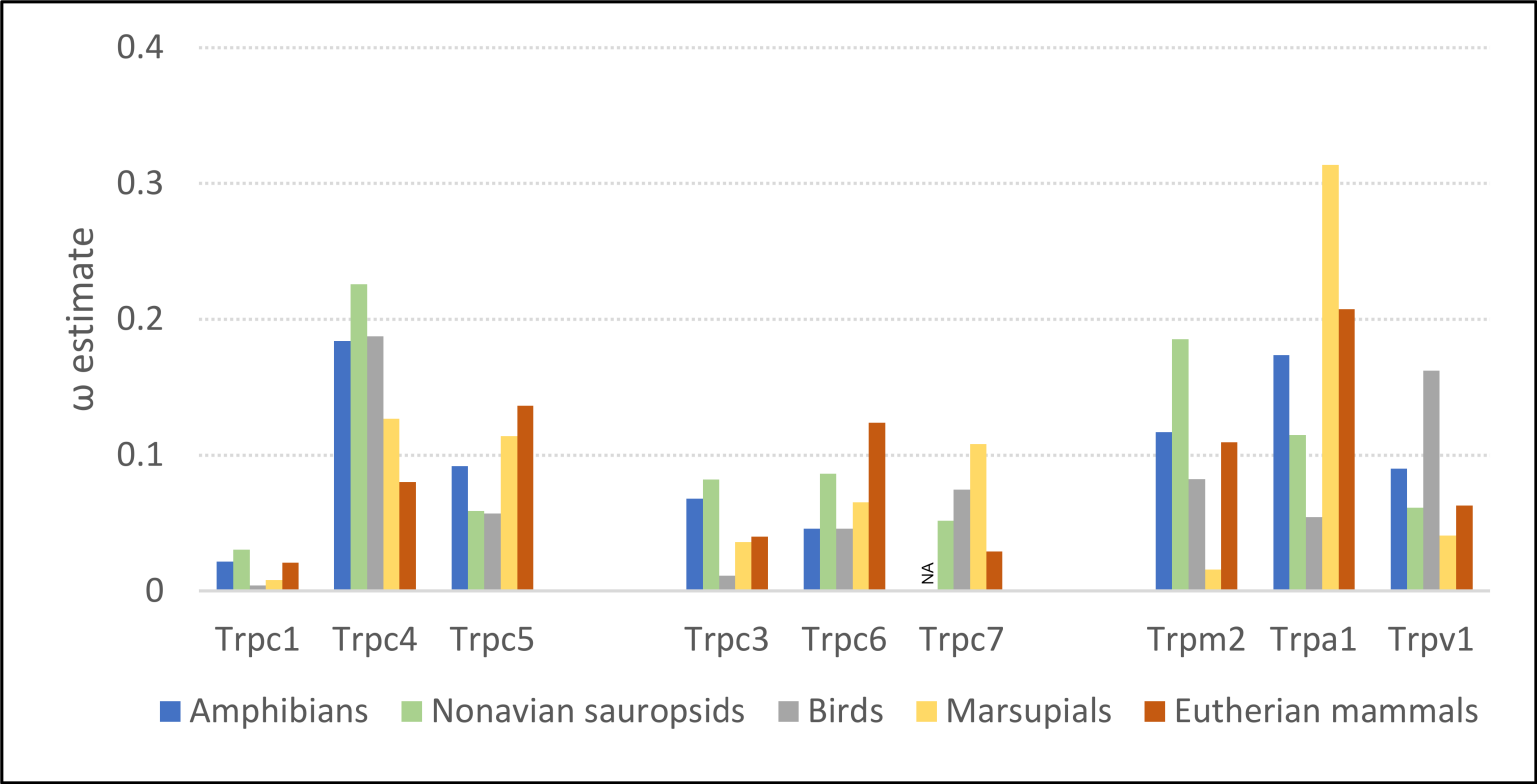
Evolutionary rates of nine transient receptor potential (Trp) ion channel genes within ectothermic and endothermic tetrapod groups. Evolutionary rate is parameterized as ω, the ratio of nonsynonymous substitutions to synonymous substitutions. NA = not applicable due to insufficient data.

Evolutionary rates of the *Trpc4* C-terminal regulatory region were similar in ectothermic clades and endothermic clades lacking NST, whereas ω was substantially lower in eutherian mammals (Fig. 2). The ω estimates for amphibians and non-avian sauropsids were 0.184 and 0.226, respectively, and 0.187 in birds. In eutherian mammals, ω was estimated to be 0.080, whereas the ω estimate for marsupials was intermediate (0.129). Evolutionary rates of other Trp genes confirm that the above pattern is specific to *Trpc4*. Only *Trpc7* had a lower ω in eutherian mammals than in the other clades, and in that comparison marsupials had the highest ω rather than ectothermic clades. Note *Trpc7* was not analyzed for amphibians as there were insufficient orthologous sequences identified for this group.

RELAX found significant evidence of intensification of selection on *Trpc4* in descendent eutherian mammals relative to the basal eutherian branch (blue branches relative to green branch in Fig. 1, *P* = 4.10E−06). A two-rate model estimated an intensification parameter of 2.71 in descendent eutherian mammals, with ω_1_ = 0.047 for a proportion of sites p_1_ = 0.933 and ω_2_ = 1 for a proportion p_2_ = 0.067 of sites. These values were ω_1_ = 0.324 and ω_2_ = 1 in the basal eutherian node (rate proportions are held constant in the model). This explicit statistical test supports the qualitative conclusion drawn from the above comparison of multiple Trp genes across multiple tetrapod groups, *i.e.,* that *Trpc4* experienced greater constraint in descendent eutherian mammals after the evolution of BAT in that clade.

### *Trpc4* evolutionary rates increased after *Ucp1* inactivation

**Figure 3:**
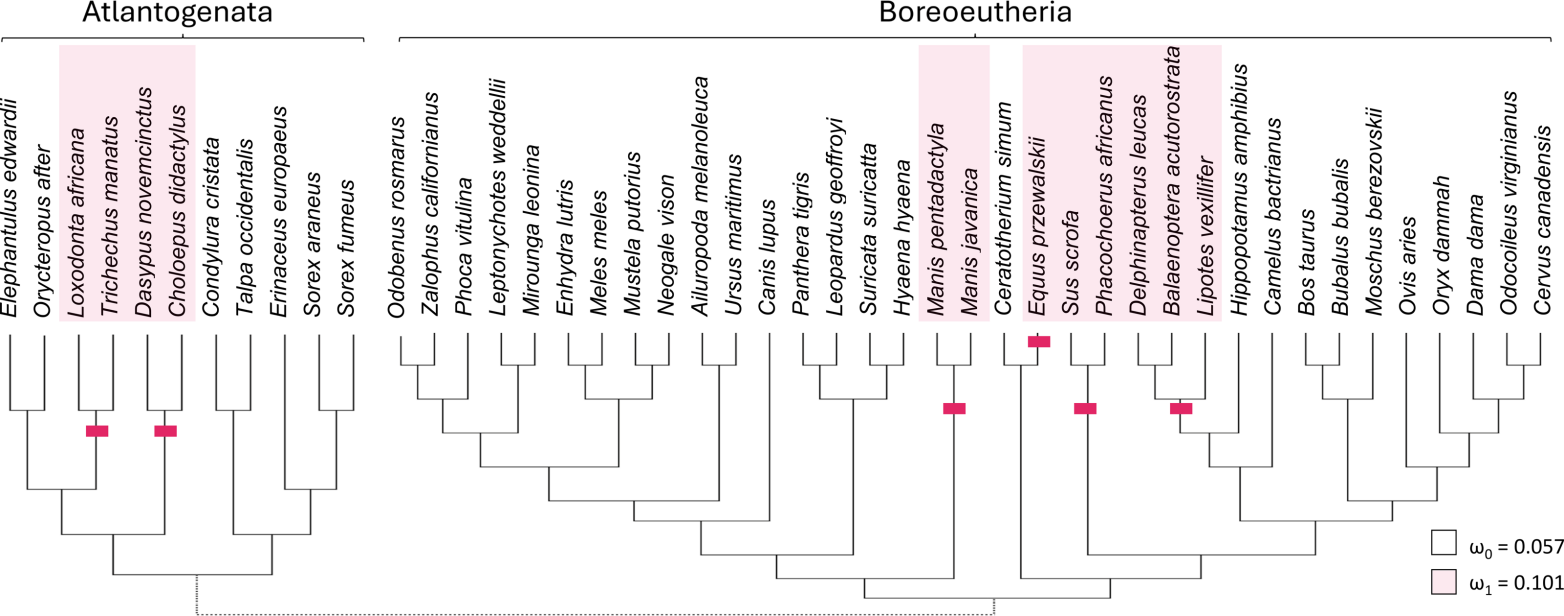
Evolutionary rates of the canonical transient receptor potential (*Trpc4*) gene are higher in eutherian clades that no longer express functional thermogenin. The red bars denote inferred pseudogenization events of the thermogenin gene *Ucp1*, taken from the literature, and red shading denotes the tip branches that were labeled as foreground (inclusive of their shared nodes) in the analysis. Evolutionary rates are modeled as the parameter ω, the ratio of nonsynonymous to synonymous substitutions along branches of a phylogeny. The values estimated for the two rate classes are designated by subscripts (ω_1_ is the overall rate in *Ucp1*-inactivated lineages compared with background rate ω_0_). A subsequent test of reduced constraint was significantly more likely than an alternative of positive selection at a subset of sites.

Taxa with presumed inactivating *Ucp1* mutations include suids, equids, cetaceans, elephantids, manatees, pangolins, armadillos, and sloths (Gaudry et al., 2017). Marking representatives of these taxa as foreground and related mammals as background (Fig. 3) yielded strong support from codeml for increased diversification rates of the TRPC4 regulatory region after *Ucp1* inactivation (FDR corrected *P* = 2.30E−05). The foreground ω was estimated to be 0.101 and the background ω was 0.0574. Note the number of cetacean taxa included in this test was reduced to avoid including very similar sequences, as short branch lengths tend to reduce the power of selection tests (Anisimova, Bielawski & Yang, 2001; Smith et al., 2015).

There are potential concerns with a global test of the effect of *Ucp1* inactivation on *Trpc4* evolutionary rates, however, as it may not be realistic to assume a single background and a single foreground rate across the entire tree (see Results below). Therefore, the data were split into two additional PAML tests to further confirm the initial result. One test was performed for Boreoeutheria and a second test for “Atlantogenata” sensu Waddell et al. (1999), *i.e.,* Xenarthra plus Afrotheria (Fig. 3). These secondary tests were both statistically significant after FDR correction (*P* = 0.0116 for Boreoeutheria and *P* = 0.00210 for Atlantogenata). Within Boreoeutheria, the background ω was estimated as 0.0631 and the foreground ω for *Ucp1*-inactivated taxa was estimated to be 0.0961. Within Atlantogenata, the background ω estimate was 0.0652 and the foreground ω esimtae for *Ucp1*-inactivated taxa was 0.119. These parameter values are similar to those estimated for the combined tree.

Relaxed selection on the regulatory region of TRPC4 after *Ucp1* inactivation, as opposed to episodic positive selection, was supported by RELAX (FDR-corrected *P* = 4.49E−06). The relaxed selection model was also significant for the Atlantogenata and Boreoeutheria alignments separately (FDR-corrected *P* = 5.93E−04 and 6.27E-04, respectively).

### Tetrapod TRPC4 sequences have diversified within a distinct, functionally uncharacterized region

**Figure 4:**
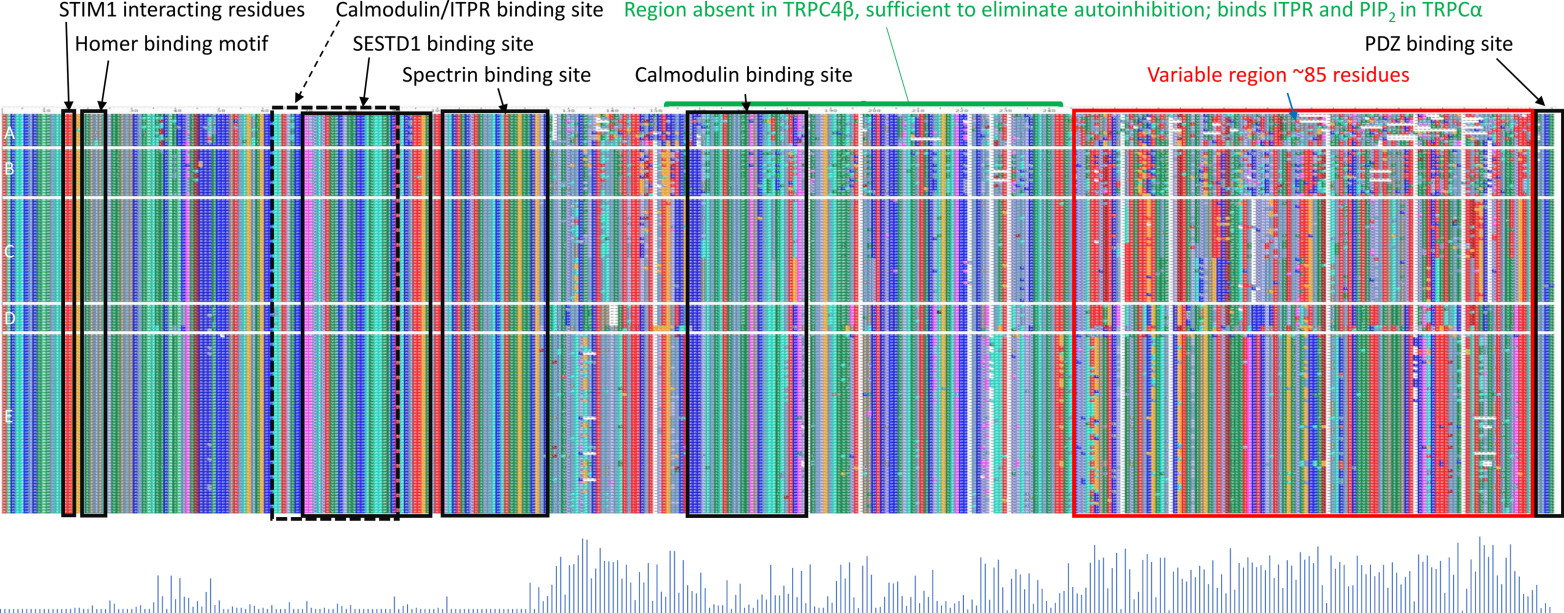
Protein sequence alignment of canonical transient receptor potential channel 4 (TRPC4) sequences from representative taxa of five tetrapod groups. Each row represents the C-terminal TRPC4 region as defined in the text, with each block in a row representing an amino-acid colorized according to biochemical properties and labeled with their standard single-letter codes (see Methods). The sequences are not intended to be legible; instead the color pattern reflects the pattern of variability within and among groups. Tetrapod groups are labeled by letters: amphibians (A), nonavian sauropsids (B), birds (C), marsupials and monotremes (D), and eutherian mammals (E). Positions of defined protein features are as described in the literature (see Methods), whereas the conserved regions of unknown function and variable regions marked in red and blue, respectively, are inferred from the alignment. As the rows represent only subsets of the total sequences available for birds and mammals, a Shannon entropy plot is shown below the aligned protein sequence that reflects overall variation among these taxa within the alignment. The Shannon entropy scale is not relevant and is omitted here; higher values correspond to greater site-level variation. STIM1 = stromal interaction molecule 1, SESTD1, SEC14 and spectrin domain containing 1; ITPR, inositol triphosphate receptor. PDZ is an initialism of three proteins in which the domain was initially described.

While a protein alignment is not informative of evolutionary rate, it can be useful to visualize how extant variation among clades relates to known functional elements within the C-terminus of TRPC4 (Fig. 4). Most of the amino-acid divergence among the tetrapod groups analyzed lies within an ∼85 amino-acid region immediately downstream of a conserved but alternatively spliced sequence (discussed further below) and immediately upstream of the terminal, conserved ‘PDZ’ domain that functions to localize TRPC channels in membranes (Mery et al., 2002). No specific protein-binding interactions have yet been localized to this region of TRPC4. In contrast, most previously characterized protein-binding sites are well conserved among all tetrapod clades examined (Fig. 4). One exception is the second calmodulin binding site identified by Tang et al. (2001), which is less conserved in the ectothermic clades and shows some variability in birds and mammals as well. The region between the second calmodulin binding site and the variable region is believed to bind ITPRs (Tang et al., 2001) but this interaction has not been further localized. These patterns indicate that the variable region upstream of the PDZ binding site is the most likely to be involved in among-clade differences in *Trpc4* function, rather than previously characterized binding sites.

### Increased evolutionary rate of *Trpc4* in bats is not generalizable to small bodied, heterothermic clades

**Figure 5:**
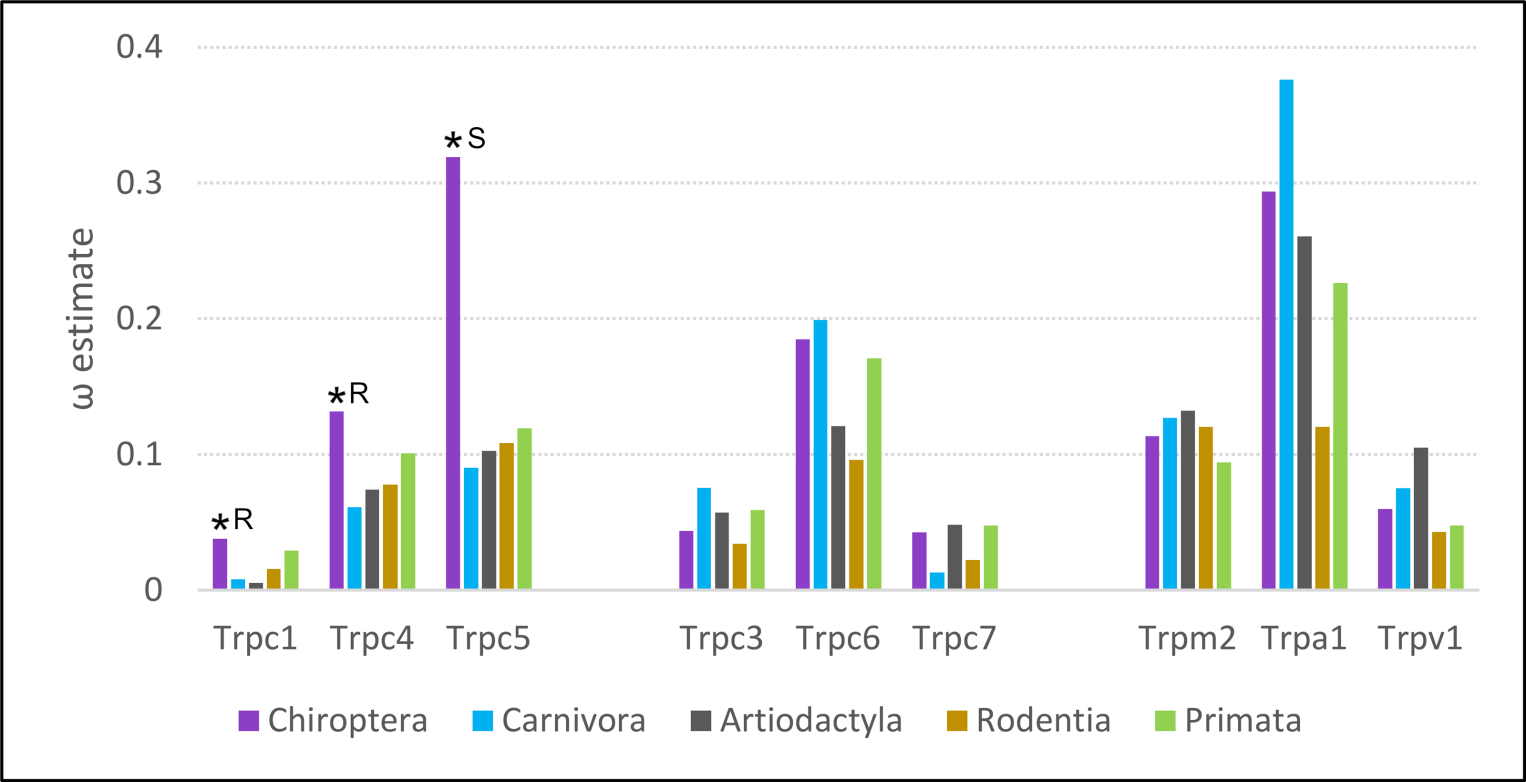
Evolutionary rates of nine transient receptor potential (Trp) ion channel genes within five mammalian orders. Evolutionary rate is parameterized as ω, the ratio of nonsynonymous substitutions to synonymous substitutions. An asterisk indicates a branch-specific test of heterogeneous selection pressure was performed for Chiroptera and was significant after adjusting for multiple tests. Follow-up tests for those significant genes yielded significant support for either relaxed constraint or positive selection, marked R and S respectively in the figure.

Of the five eutherian orders analyzed, the evolutionary rate of *Trpc4* was highest in bats (Fig. 5). Bats also had the highest evolutionary rates for the *Trpc4*-interacting homologs *Trpc5* and *Trpc1*. Estimates of ω for all three genes were significantly higher in bats than background, represented by outgroups Carnivora and Artiodacytla, in codeml branch tests of evolutionary rate (FDR-adjusted *P* = 9.64E−08 for *Trpc4*, *P* = 2.092E−13 for *Trpc5*, and *P* = 0.00361 for *Trpc1*). Bats did not have higher evolutionary rates for Trpc genes that do not interact with *Trpc4* in neurons (*Trpc3*, *Trpc6*, and *Trpc7*), nor did they have elevated rates for the other Trp genes implicated in temperature perception and BAT modulation (*Trpm2*, *Trpv2*, and *Trpa1*). In contrast to bats, ω was estimated to be lower in rodents than in primates for eight of nine genes evaluated, including for *Trpc4*. Thus, while bats show increased diversification rates of *Trpc4* as well as the interacting genes *Trpc5* and *Trpc1* since their divergence from other mammals, the pattern is not generalizable to other clades exhibiting high variation in Tb.

Analysis with HyPhy favored an interpretation of relaxed selection on *Trpc4* and *Trpc1* in bats and positive diversifying selection on *Trpc*5 in bats. RELAX supported relaxed selection on *Trpc4* and *Trpc1* (FDR-corrected *P* = 1.590E−11 and 9.13E-4, respectively) whereas the positive selection model of BUSTED was not significant for either. Conversely, the BUSTED model of positive selection within bats was significant for *Trpc5* at *P* = 0.0459, and the RELAX model found evidence of intensified selection on that gene in bats (4.890E-12).

### Changes in GC content and alternative splicing in eutherian *Trpc4* genes

Inspection of *Trpc4* alignments revealed individual lineages within multiple orders with a high number of amino-acid substitutions, such as camelids and heteromyid rodents (Fig. S2). *Ad-hoc* branch-specific tests of evolutionary rate were not significant when these lineages were marked as foreground and related taxa marked as background, however, apparently because both nonsynonymous and synonymous substitutions were elevated in the foreground taxa (File S2). This pattern of elevated substitution rates was driven by localized shifts in GC content within the terminal exon only in these taxa, rather than at the gene or higher level (File S3). These high-GC exons are computationally inferred by NCBI to contain CpG islands, which is unusual given that CpG islands are typically upstream of rather than within coding sequence, due to their strong association with transcription initiation and their intrinsically high mutation rate (Deaton & Bird, 2011; Angeloni & Bogdanovic, 2021).

Cornman (2024) also noted repeated shifts in GC content of *Trpc4* in several bat taxa, but in those cases the shift was Mb-scale and encompassed more than a dozen genes, and therefore was more similar to ‘isochore’ variation (Bernaola-Galván et al., 2023). To assess whether GC shifts within some bat clades biased the estimation of ω and the statistically significant branch test for *Trpc4*, the test was repeated with high GC taxa removed (File S3). The *P*-value for this modified test was also highly significant (FDR corrected *P* = 2.26E−11), demonstrating that the increased evolutionary rate of *Trpc4* in bats is not an artifact of shifts in GC composition or extreme synonymous rate variation (Ratnakumar et al., 2010; Wisotsky et al., 2020).

The recurrence of GC peaks within the terminal exon of some *Trpc4* genes is of uncertain significance, and may be incidental to the regulation of nearby genes. However, as CpG and GC levels also influence alternative splicing (Zhang, Kuo, Chen, 2011) and intron retention (Monteuuis et al., 2019), these compositional shifts may instead relate to the alternative splicing of the “beta” isoform of TRPC4 (TRPC4β, see Fig. 3). TRPC4β is generated by excision of an intron that is otherwise read through in the predominant “alpha” variant, TRPC4α. The two TRPC4 variants form functionally distinct channels in cell culture and have different regulatory responses (Schaefer et al., 2002; Otsuguro et al., 2008), but the phenotypic relevance of these alternative isoforms in tissues remains uncertain. Schaefer et al. (2002) found that TRPC4α expressed in cultured cells was autoinhibited whereas TRPC4β was not, implying a regulatory interaction within the C-terminal exon of TRPC4α by unidentified activators. TRPC4α also binds PIP_2_ within the region excised from TRPC4β (Cooley et al., 2014).

Interestingly, the TRPC4β isoform appears to be derived in eutherian mammals, as the splicing event is neither annotated nor evident in RNA alignments displayed in NCBI’s Gene database (Brown et al., 2015) for western clawed frog, garter snake, chicken, platypus, or opossum, and the canonical splicing signals (Lee & Rio, 2015) are absent in those groups (Fig. S3). Subsequent loss of splice signals also appears relatively common in eutherian mammals based on the aligned orthologs, including some species with high GC content within *Trpc4* noted above (Fig. S4). Thus, the beta isoform appears to have originated at approximately the same time UCP1 evolved thermogenic properties, and both show subsequent evolutionary lability. Whether TRPC4β influences NST thermoregulation is entirely speculative at present, however.

## Discussion

NST is a key mammalian innovation but exhibits evolutionary lability as mammalian taxa have adapted to diverse environments and niches (Rodríguez-Serrano & Bozinovic, 2009; Boyles et al., 2013). For example, taxa tolerate different ranges of Tb variation during normal activity and differ in how much Tb can be increased by NST (*e.g.*, Nespolo, Opazo & Bozinovic, 2001; Gębczyński & Taylor, 2004), although species also exhibit substantial plasticity in these traits as well (Van Sant & Hammond, 2008 (Panaino et al., 2023). NST may also be a component of thermoregulatory strategies such as torpor and hibernation, which have distinct endocrinal and metabolic drivers (Hashimoto et al., 2002). Thus, the genetic mechanisms by which NST responses are modulated within and across taxa are likely diverse. Yet the discovery that *Trpc4* is required for mouse NST (Zhou et al., 2023) combined with the observation of high diversification rates in the regulatory region of TRPC4 within bats (Cornman, 2024) suggested a hypothesis that relatively simple sequence changes in TRPC4 could underlie aspects of thermoregulatory adaptation in that clade and perhaps in mammals generally.

In this study, multiple comparisons of evolutionary rate were made to assess whether evolutionary change in the regulatory region of TRPC4 was consistent with this hypothesis, which was supported for mammals generally. First, the evolutionary rate of *Trpc4* substantially increased after the divergence of eutherian mammals from non-eutherian ancestors coincident with the evolution of BAT (Fig. 1), although no evidence of a rate class ω >1 was found with aBSREL. *Trpc4* subsequently became significantly more constrained in descendent Eutheria relative to the basal branch. Moreover, *Trpc4* evolutionary rates are substantially lower in eutherian orders than in all clades lacking NST, a pattern not mirrored in any other Trp gene analyzed (Fig. 2), including genes that control Tb homeostasis in warm-sensitive neurons but are not known to function in the induction of NST (Song et al., 2016; Kamm et al., 2021). Additionally, evolutionary rates of *Trpc4* significantly increased under relaxed selection in eutherian lineages that later lost thermogenin (UCP1), a major component of NST (Fig. 3). While these results are correlative and no specific amino-acid substitutions in TRPC4 have yet been demonstrated to modify NST regulation by WSNs, the data are nonetheless consistent across multiple independent predictions.

In contrast, no simple relation was identified between heterothermy in small-bodied mammals and *Trpc4* evolutionary rates (Fig. 5). *Trpc4* as well as *Trpc5* and *Trpc1* diversified at significantly higher rates in bats than in outgroups, indeed higher than all other eutherian groups analyzed. In contrast, *Trpc4* did not have a higher evolutionary rate in rodents compared with primates. Thus, the increased diversification of a suite of Trpc genes that interact in neurons appears to be distinctive of bats and not a recurring pattern in small-bodied, heterothermic eutherian mammals. In fact, *Trpc4* and *Trpc1* appear to be under relaxed selection rather than diversifying selection in bats, suggesting that regulation of NST by WSNs may be less constrained in species employing frequent daily torpor or prolonged bouts of heterothermy while not actively foraging, as is common in this clade (Stawski, Willis & Geiser, 2014). Alternatively, given the support for positive selection on *Trpc5* in bats and the physical interaction of these gene products in heteromeric channels, it is possible that the increased evolutionary rates of *Trpc4* and *Trpc1* are compensatory to adaptive changes in TRPC5 (*e.g.*, Haag & Molla, 2005; Haag, 2007).

*Trpc4*, *Trpc5, and Trpc1* function in diverse tissues and contexts, often as part of a network of overlapping signals that are integrated at the cellular level (Chen et al., 2020), such that many phenotypic targets of selection could be imagined. Even so, a particularly interesting alternative to thermoregulation is the role of these genes in spatial memory, which is a key aspect of bat behavior (Geva-Sagiv et al., 2015). Triple knockout mice lacking *Trpc4*, *Trpc5*, and *Trpc1* in hippocampal neurons have deficits in spatial memory and learning (Bröker-Lai et al., 2017). Spatial memory is also impaired in mice that are deficient in TRPC1 alone, either genetically or *via* pharmacological inhibition (Lepannetier et al., 2018). Furthermore, *Trpc4* and *Trpc5* are particularly densely expressed in pyramidal neurons of the hippocampus (Fowler et al., 2007), which have long been recognized as key contributors to spatial memory (Burgess, Maguire & O’Keefe, 2002; Geva-Sagiv et al., 2015), and the TRPC4β isoform has been shown to influence the dendritic morphology of hippocampal neurons (Jeon et al., 2013).

Other behavioral phenotypes may also be relevant, as *Trpc4* has been linked to autism in multiple human association studies (Gupta et al., 2023; Drago, Calabro & Crisafulli, 2024) and a mouse *Trpc4* knockout model shows strong deficits in sociality (Seo et al., 2024). Both *Trpc4* and *Trpc5* nulls independently diminish anxious or fearful behaviours mediated by the amygdala (Riccio et al., 2009; Riccio et al., 2014), as do pharmacological inhibitors of their protein products (Just et al., 2018). Spontaneous human variants in *Trpc5*, including partial deletions and nonsynonymous substitutions, are associated with obesity, food hoarding, and impaired sociality in clinical cases and in UK Biobank data (Li et al., 2024). These phenotypes are strikingly recapitulated in mouse models of some of these human variants (Li et al., 2024). Interestingly, two of the nonsynonymous substitutions identified by Li et al. (2024) in the C-terminal regulatory region of human TRPC5 influenced protein decay rates in cell culture, suggesting a possible functional relevance of positive selection on these regions.

While it is increasingly feasible to express modified alleles in cell culture or in genetic model organisms, functional assessment of diverse wild-type alleles in an ecologically relevant context is more challenging, particularly as the molecular pathways that link *Trpc4* to the initiation or maintenance of NST are not yet delineated. Nonetheless, combinatorial knock-in studies in mice could provide insights into the ecological significance of sequence divergence in TRPC1/TRPC4/TRPC5 heteromeric channels in bats, as might pharmacological treatment of bats with targeted inhibitors of those proteins (Rubaiy et al., 2017; Just et al., 2018). Comparative assessments of the relative expression of Trpc homologs at the transcript and protein levels (*e.g.*, Li et al., 2022; Lv et al., 2023) could elucidate functional aspects of ecological differentiation between closely related species that are not manifested at the codon level.

### Caveats and future directions

Codon models are inherently conservative measures of past diversifying selection and can be sensitive to the evolutionary scale investigated (tree length), variable mutation rates, and incorrect inferences of phylogeny or orthology (Anisimova, Bielawski & Yang, 2001; Smith et al., 2015; Wisotsky et al., 2020; Álvarez-Carretero, Kapli & Yang, 2023). Codon models must also reconstruct unobserved ancestral sequences, although parameter estimation is not dependent on a single reconstruction but instead integrates across likelihood-weighted alternatives (Anisimova, 2012; Yang, 1998). To minimize the impact of these uncertainties, alignments were generated with as many taxa as possible at the time of data access while also maintaining balance among clades and dereplicating very similar sequences. Phylogenetically deep alignments were avoided where possible to reduce alignment ambiguity and gaps. To further support the relevance of the findings for *Trpc4*, additional genes of the same protein superfamily with potentially overlapping functions were also evaluated, facilitated by their broadly conserved secondary and tertiary structure.

An additional caveat is that large increases in ω on a branch of interest can be consistent with both positive selection and relaxed purifying selection. Only ω values substantially greater than one are unambiguously attributable to positive selection, yet values this high across many codons of a gene may not be realistic. For example, aBSREL did not find evidence of positive selection on *Trpc4* in the basal branch of eutherian mammals, but the method has low power to detect weaker episodes of positive selection, *e.g.*, ω < 4 over fewer than 20% of sites (refer to Fig. 1 of Smith et al., 2015). Furthermore, codeml, BUSTED, and RELAX branch models require that a background or reference group be specified, which adds contingency to the results.

This study focused on the C-terminal region of TRPC4, as the N-terminal ankyrin repeats and central transmembrane folds are much more conserved (Fig. S5). However, this does not imply that the latter regions have no role in adaptation at the protein level. In fact, Yan et al. (2022) identified substitutions in the N-terminal region of TRPA1 that were associated with heat tolerance in a snake. Nonetheless, the low total number of substitutions in those regions do not favor the detection of diversifying selection with codon substitution models (Anisimova, Bielawski & Yang, 2001).

While this study benefits from the increasing availability of orthologous coding sequences across the vertebrate tree of life, currently annotated genomes limit comparisons within mammals to approximately order level in practice. At this phylogenetic level, useful distinctions are possible with respect to variation in Tb, yet comparing *Trpc4* evolutionary rates at finer phylogenetic scales and with explicit physiological covariates such as thermal scope (Boyles et al., 2013) could identify associations not evident in this study. Ultimately, however, functional studies would be needed to demonstrate a relationship between specific amino-acid changes and protein function (*e.g.*, Keipert et al., 2024).

## Conclusions

*Trpc4* was previously shown by a genetic screen to be a required gene for the regulation of NST by WSNs in mice. This study provided evidence from molecular evolutionary analyses that the regulatory regions of the TRPC4 protein evolved under episodic positive and purifying selection regimes in eutherian mammals consistent with selection on NST. These selection patterns implicate a discrete region of C-terminal sequence in the recruitment of TRPC4 into the regulation of NST, which should be a useful target of functional studies on NST. While TRPC4 may be a target of ongoing mammalian adaptation to diverse thermal niches, the data do not support a general increase in TRPC4 divergence rates among small-bodied mammalian clades that show greater phenotypic divergence in heterothermy than do larger-bodied outgroups.

## Supplemental Information

10.7717/peerj.19697/supp-1 Supplemental Information 1 Control file settings and input files for PAML analyses in a ZIP-compressed folder Required and optional parameters that are read by the codeml package of PAML. This file is for information only, an explicit value must be selected for Model and the dummy file names must be replaced with explicit paths to function. The sequence and guide tree files are in standard formats (FASTA and Newick, respectively).

10.7717/peerj.19697/supp-2 Supplemental Information 2 Summarized output from PAML for each comparison and P-values for each statistical comparison Outputs are grouped by analysis on each page of the worksheet.

10.7717/peerj.19697/supp-3 Supplemental Information 3 Increased GC content and novel CpG islands in the terminal exon of Trpc4 in selected eutherian mammals relative to related taxa Images are taken from the Genome Browser utility of the National Center for Biotechnology (NCBI), which displays genome features calculated by NCBI. Each panel shows the exon-intron structure of the gene model (top ideogram), the percent GC content in a 50-bp sliding window (middle ideogram), and computationally classified CpG islands. GC content is shown in the range 25-75%; windows that exceed this range are marked by red squares. The red circles identify the region of interest within the terminal exon that shows parallel changes in composition across the four examples shown in the document. Images acquired 1/15/2024 from genome browsers available at https://ncbi.nlm.nih.gov.

10.7717/peerj.19697/supp-4 Supplemental Information 4 Neighbor-joining dendrogram of six canonical transient receptor potential (Trpc) genes from nine tetrapod taxa support the inferred orthogroups The amino-acid dendrogram was generated from complete protein sequences as described in the text; the accession number of each protein prediction is shown. Note *Trpc7* is not annotated in the western clawed frog (*Xenopus tropicalis*).

10.7717/peerj.19697/supp-5 Supplemental Information 5 Examples of clades with high numbers of private amino-acid substitutions within the C-terminal region of canonical transient receptor potential channel protein 4 (TRPC4), highlighted by red boxes The standard letter code for each amino acid is shown for the first sequence in each alignment; subsequent sequences are represented as a dot if they match the first sequence or by the standard letter code if the differ from the first sequence. This scheme serves to highlight the overall distribution of variation rather than specific changes at particular sites. Background color of each residue is based on the default biochemical similarity scheme used in BioEdit (Hall TA. 1999. BioEdit: a user-friendly biological sequence alignment editor and analysis program for Windows 95/98/NT. In: *Nucleic acids symposium series*. [London]: Information Retrieval Ltd., c1979-c2000., 95–98.). In panel A, camelids are highlighted relative to other Artiodactyla. In panel B, the genera *Perognathus* and *Dipodomys* are highlighted relative to other Rodentia. Any use of trade, firm, or product names is for descriptive purposes only and does not imply endorsement by the U.S. Government.

10.7717/peerj.19697/supp-6 Supplemental Information 6 No evidence of the canonical transient receptor potential 4 beta (TRPC4 β) isoform splice signals in well-annotated genomes of non-placental mammals Alignments show the intron sequence that is spliced from the mRNA of TRPC4 β but is retained in TRPC4 α . The boxes highlight canonical intron features that are all present in the eutherian mammals shown but not all present in the other tetrapod taxa. The red box indicates the donor site, the canonical sequence of which is “GT”. The green box indicates the acceptor site, the canonical sequence of which is “AG”. The blue box indicates the pyrimidine tract, upstream of which is the canonical branch site “A”. Sequences that have all three canonical signals are marked with green circles next to their titles, otherwise with red.

10.7717/peerj.19697/supp-7 Supplemental Information 7 Loss of splice signals for the canonical transient receptor potential 4 beta (TRPC4 β) isoform in eutherian mammals The boxes highlight canonical splice signals; sequences that have all canonical signals are marked with green circles next to their titles, otherwise with red. The red box indicates the donor site, the canonical sequence of which is “GT”. The green box indicates the acceptor site, the canonical sequence of which is “AG”. The blue box indicates the pyrimidine tract, upstream of which is the canonical branch site “A”. Examples are shown from Chiroptera are shown in panel A, from Carnivora in panel B, and from Artiodactyla in panel C. Loss of splice signals does not demonstrate that alternative splicing does not occur in a noncanonical fashion or from newly arisen signals nearby in the primary transcript. Note the alignments wrap to two lines in each panel.

10.7717/peerj.19697/supp-8 Supplemental Information 8 Shannon entropy plot of amino-acid variation in 214 aligned canonical transient receptor potential channel 4 (TRPC4) proteins from mammals The red box represents the C-terminal region analyzed in the text.

## Additional Information and Declarations

### Data Availability

The following information was supplied regarding data availability: The input files and program settings for PAML analyses are available in the Supplemental Files.
