# Supplementary material for: Molecular evolution of TRPC4 regulatory sequences supports a role in mammalian thermoregulatory adaptation
Source: PeerJ. 2025 Jul 8;13:e19697. doi: 10.7717/peerj.19697 (PMC12248226; doi:10.7717/peerj.19697)

Supplemental File S3. Increased GC content and novel CpG islands in the terminal exon of canonical transient receptor potential 4 (*Trpc4*) in selected eutherian mammals relative to related taxa. Images are taken from the Genome Browser utility of the National Center for Biotechnology (NCBI), which displays genome features calculated by NCBI. Each panel shows the exon-intron structure of the gene model (top ideogram), the percent GC content in a 50-bp sliding window (middle ideogram), and computationally classified CpG islands. GC content is shown in the range 25-75%; windows that exceed this range are marked by red squares. The red circles identify the region of interest within the terminal exon that shows parallel changes in composition across the four examples shown in the document. Images acquired 1/15/2024. The genome browsers from which the images were captured can be accessed at https://ncbi.nlm.nih.gov.

Example 1: *Otolemur garnettii* and *Propithecus coquereli* (Primates)

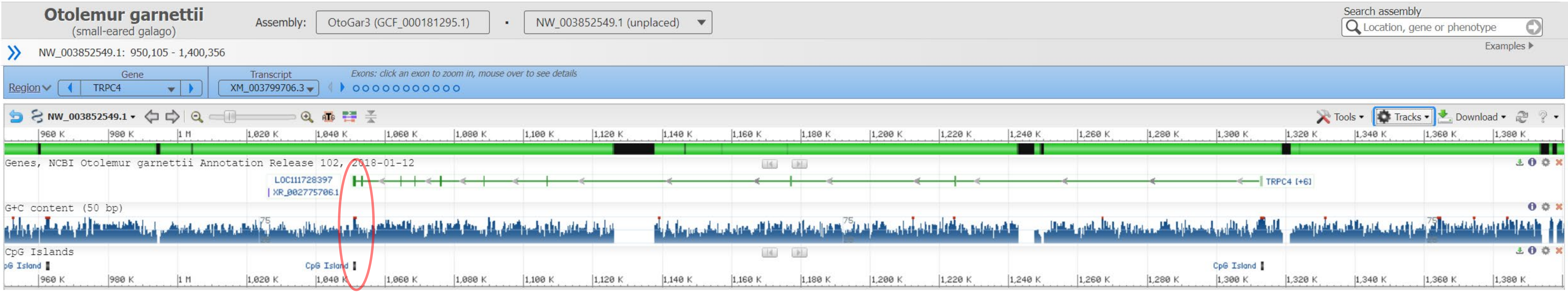

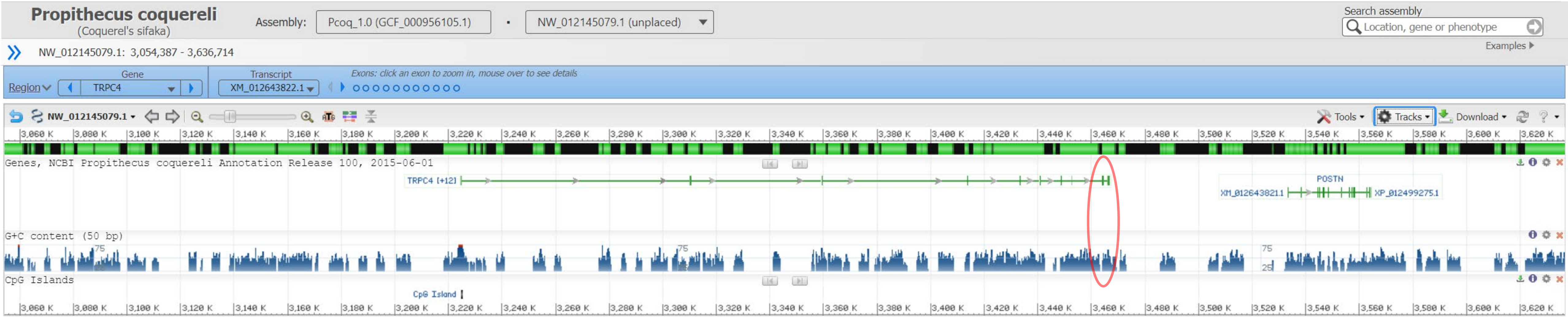

Example 2: *Dipodomys spectabilis* and *Castor canadensis* (Rodentia)

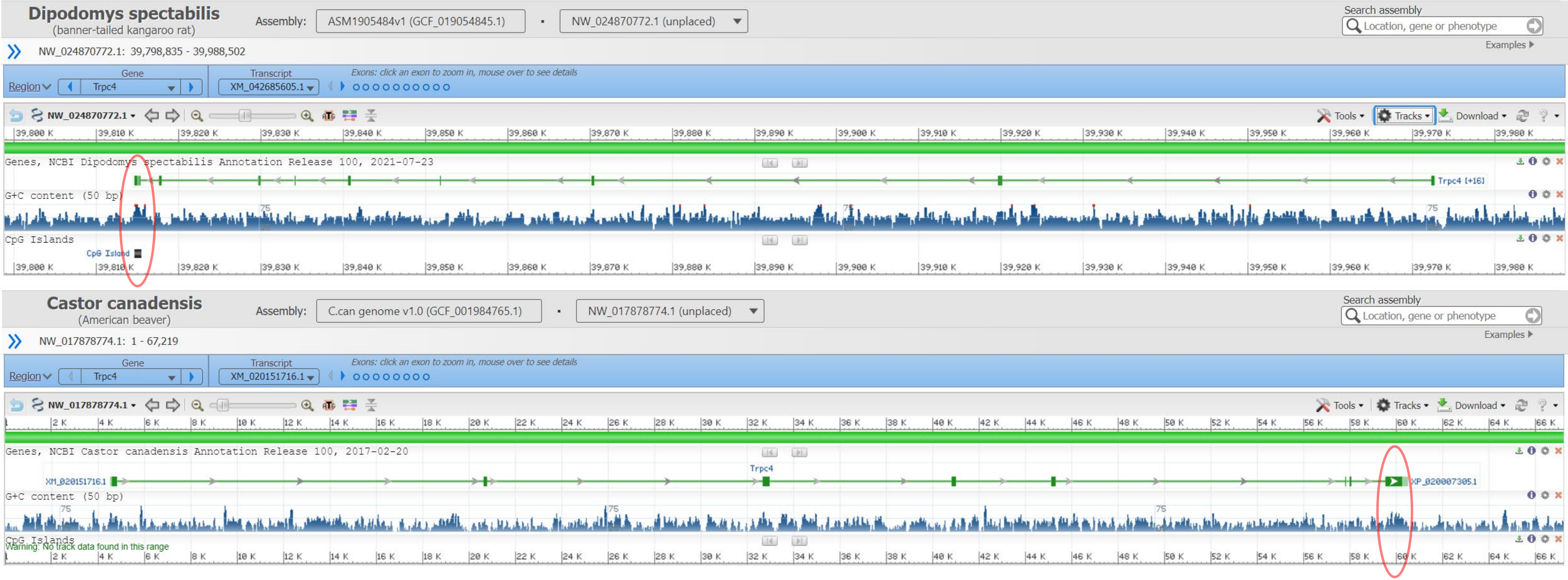

Example 3: *Manis javanica* (Pholidota) and *Canis lupus* (Carnivora)

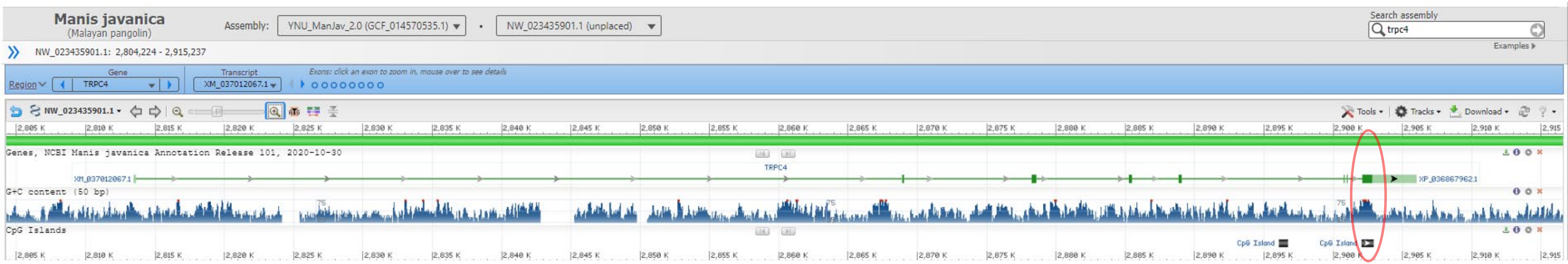

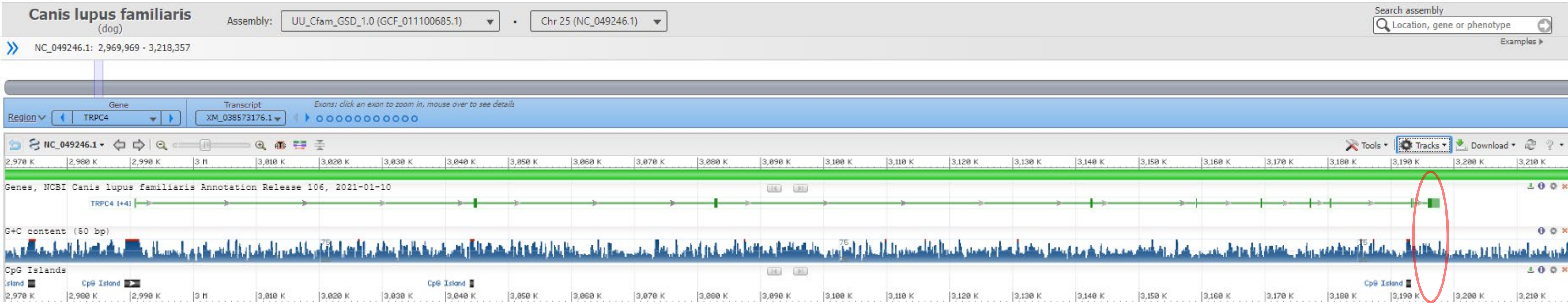

Example 4: *Rousettus aegyptiacus* and *Artibeus jamaicensis* (Chiroptera)

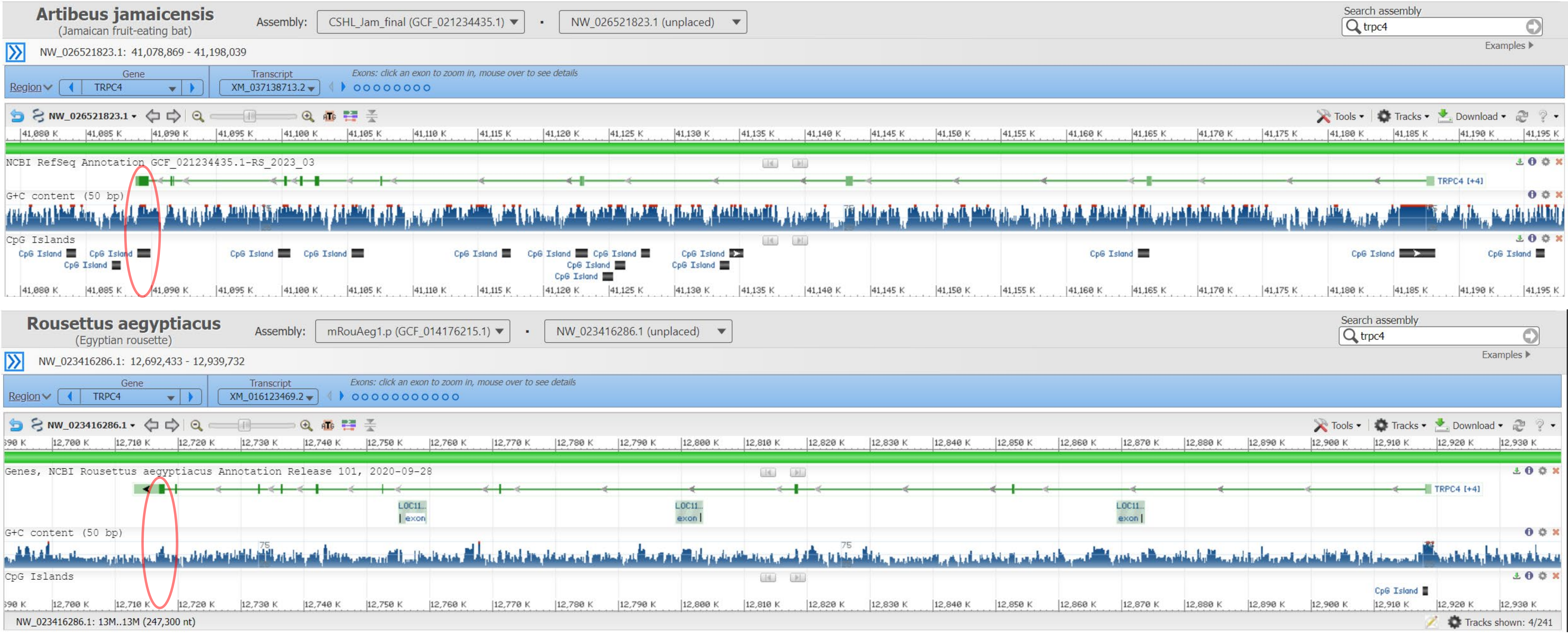

Supplement: Supplemental Information 3 — Images are taken from the Genome Browser utility of the National Center for Biotechnology (NCBI), which displays genome features calculated by NCBI. Each panel shows the exon-intron structure of the gene model (top ideogram), the percent GC content in a 50-bp sliding window (middle ideogram), and computationally classified CpG islands. GC content is shown in the range 25-75%; windows that exceed this range are marked by red squares. The red circles identify the region of interest within the terminal exon that shows parallel changes in composition across the four examples shown in the document. Images acquired 1/15/2024 from genome browsers available at https://ncbi.nlm.nih.gov. [file peerj-13-19697-s003.pdf]
