# Supplementary figures and images for: Molecular evolution of TRPC4 regulatory sequences supports a role in mammalian thermoregulatory adaptation
Source: PeerJ. 2025 Jul 8;13:e19697. doi: 10.7717/peerj.19697 (PMC12248226; doi:10.7717/peerj.19697)

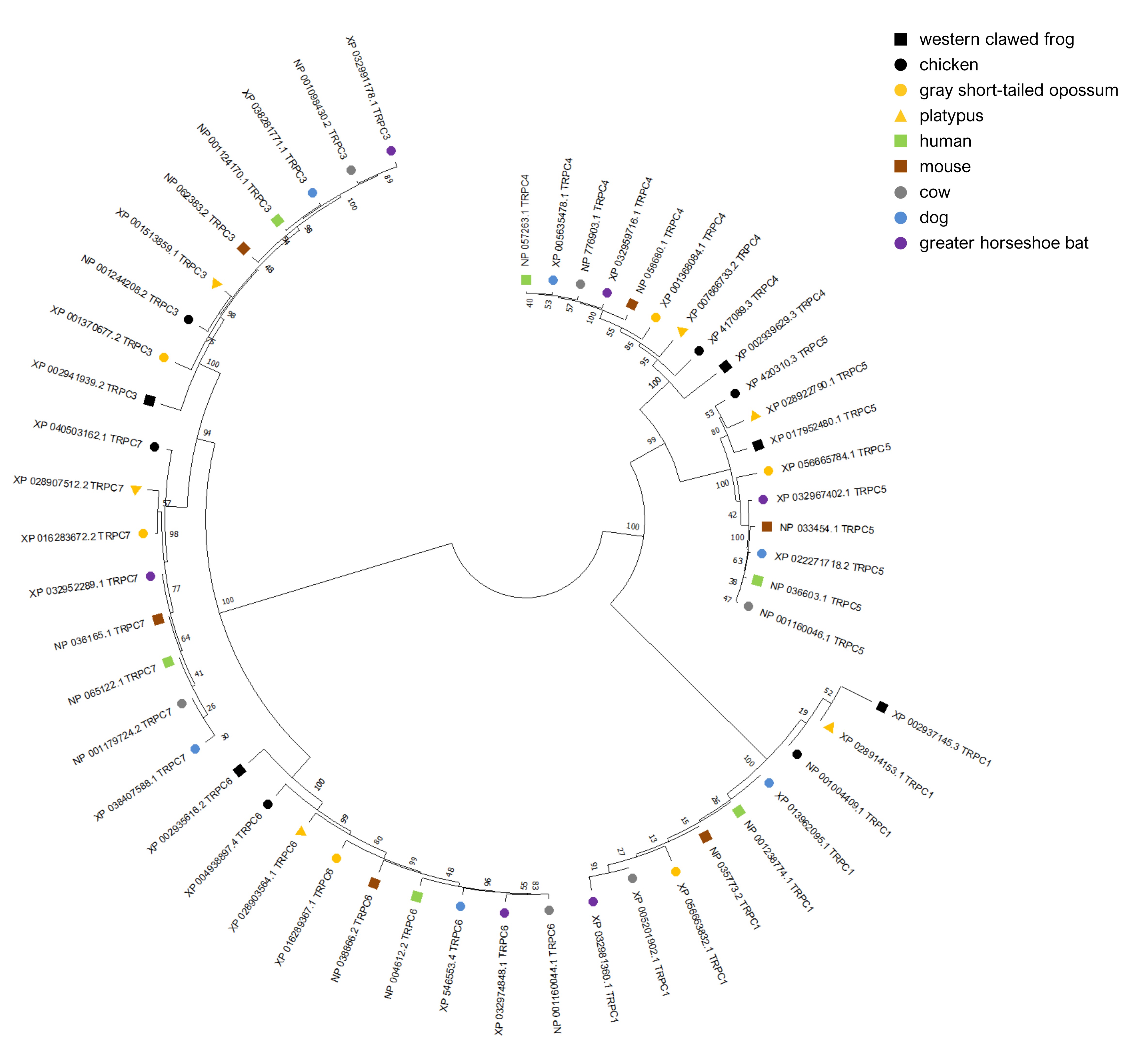

Supplement: Supplemental Information 4 — The amino-acid dendrogram was generated from complete protein sequences as described in the text; the accession number of each protein prediction is shown. Note Trpc7 is not annotated in the western clawed frog (Xenopus tropicalis). [file peerj-13-19697-s004.png]

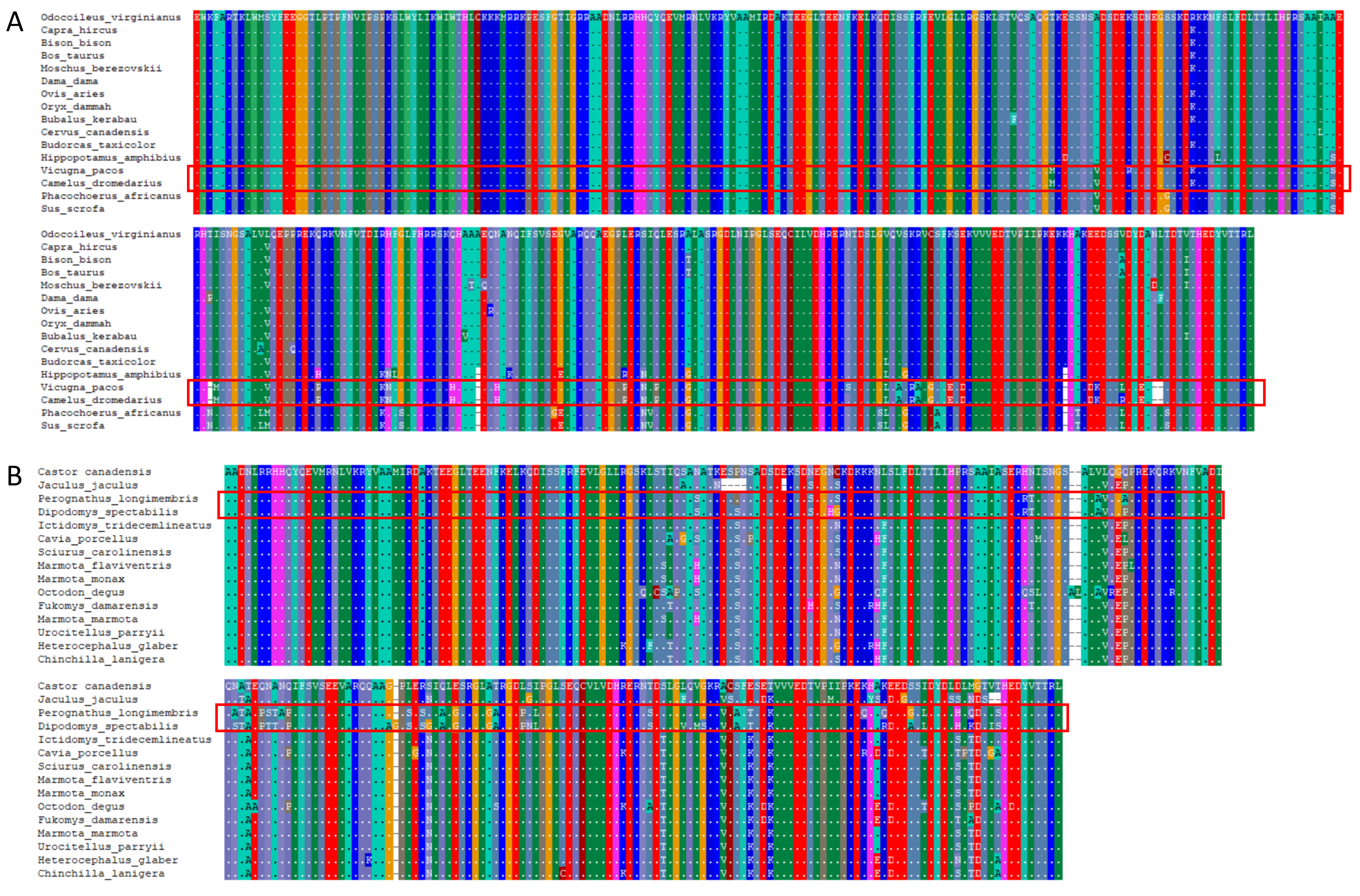

Supplement: Supplemental Information 5 — The standard letter code for each amino acid is shown for the first sequence in each alignment; subsequent sequences are represented as a dot if they match the first sequence or by the standard letter code if the differ from the first sequence. This scheme serves to highlight the overall distribution of variation rather than specific changes at particular sites. Background color of each residue is based on the default biochemical similarity scheme used in BioEdit (Hall TA. 1999. BioEdit: a user-friendly biological sequence alignment editor and analysis program for Windows 95/98/NT. In: Nucleic acids symposium series. [London]: Information Retrieval Ltd., c1979-c2000., 95–98.). In panel A, camelids are highlighted relative to other Artiodactyla. In panel B, the genera Perognathus and Dipodomys are highlighted relative to other Rodentia. Any use of trade, firm, or product names is for descriptive purposes only and does not imply endorsement by the U.S. Government. [file peerj-13-19697-s005.png]

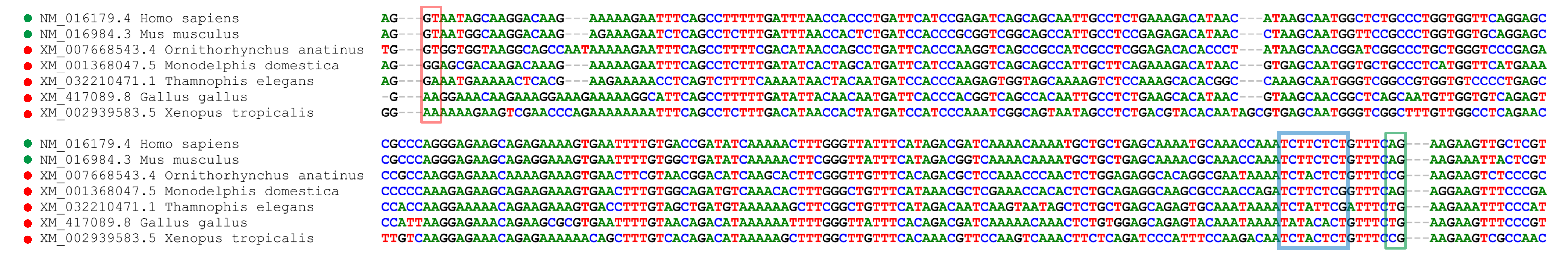

Supplement: Supplemental Information 6 — Alignments show the intron sequence that is spliced from the mRNA of TRPC4 β but is retained in TRPC4 α . The boxes highlight canonical intron features that are all present in the eutherian mammals shown but not all present in the other tetrapod taxa. The red box indicates the donor site, the canonical sequence of which is “GT”. The green box indicates the acceptor site, the canonical sequence of which is “AG”. The blue box indicates the pyrimidine tract, upstream of which is the canonical branch site “A”. Sequences that have all three canonical signals are marked with green circles next to their titles, otherwise with red. [file peerj-13-19697-s006.png]

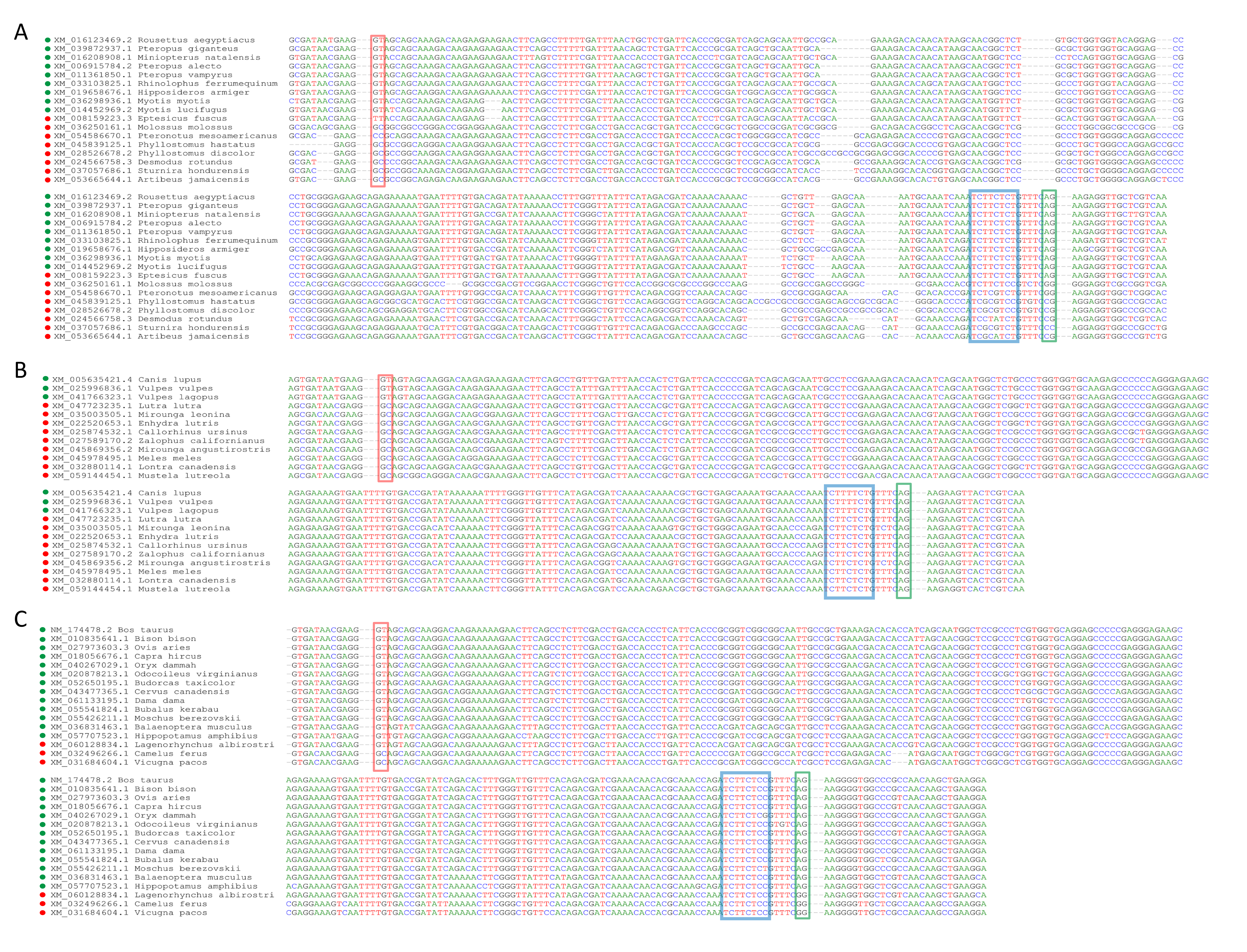

Supplement: Supplemental Information 7 — The boxes highlight canonical splice signals; sequences that have all canonical signals are marked with green circles next to their titles, otherwise with red. The red box indicates the donor site, the canonical sequence of which is “GT”. The green box indicates the acceptor site, the canonical sequence of which is “AG”. The blue box indicates the pyrimidine tract, upstream of which is the canonical branch site “A”. Examples are shown from Chiroptera are shown in panel A, from Carnivora in panel B, and from Artiodactyla in panel C. Loss of splice signals does not demonstrate that alternative splicing does not occur in a noncanonical fashion or from newly arisen signals nearby in the primary transcript. Note the alignments wrap to two lines in each panel. [file peerj-13-19697-s007.png]

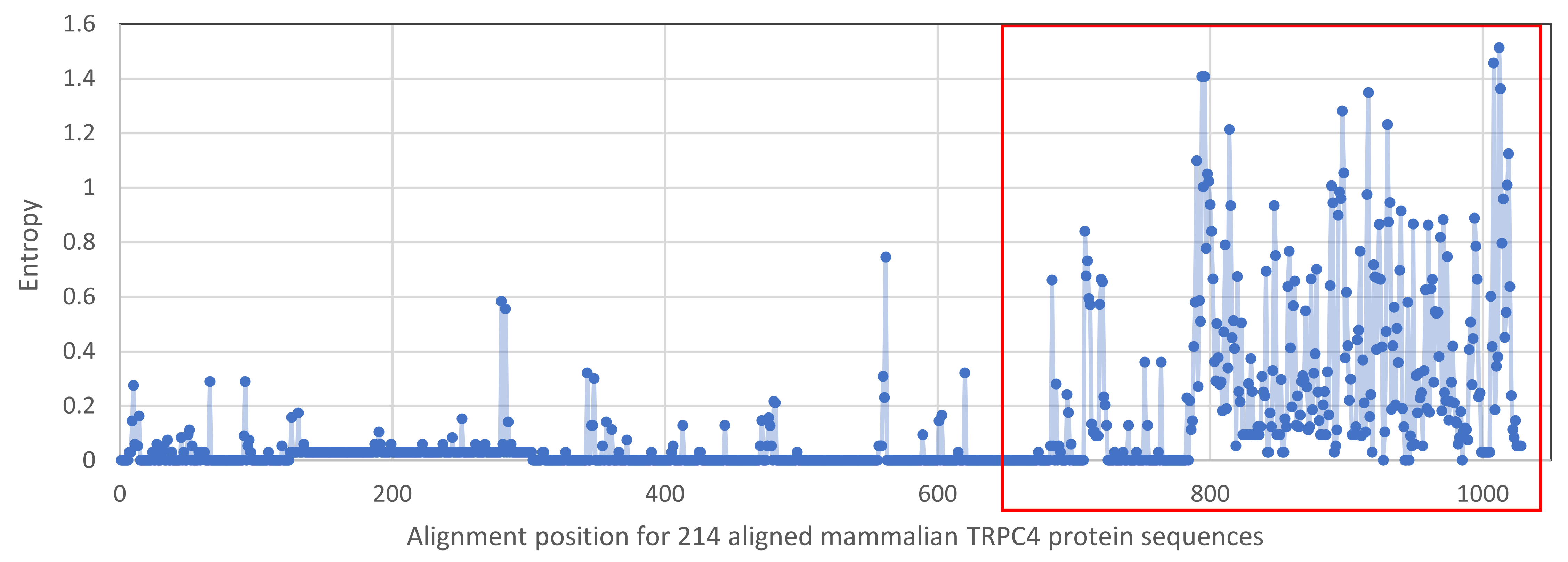

Supplement: Supplemental Information 8 — The red box represents the C-terminal region analyzed in the text. [file peerj-13-19697-s008.png]
